# Conservation of the abscission signaling peptide IDA during Angiosperm evolution: withstanding genome duplications and gain and loss of the receptors HAE/HSL2

**DOI:** 10.3389/fpls.2015.00931

**Published:** 2015-10-30

**Authors:** Ida M. Stø, Russell J. S. Orr, Kim Fooyontphanich, Xu Jin, Jonfinn M. B. Knutsen, Urs Fischer, Timothy J. Tranbarger, Inger Nordal, Reidunn B. Aalen

**Affiliations:** ^1^Section for Genetics and Evolutionary Biology, Department of Biosciences, University of OsloOslo, Norway; ^2^UMR Diversité et Adaptation et Développement des Plantes, Institut de Recherche pour le DéveloppementMontpellier, France; ^3^Department of Forest Genetics and Plant Physiology, Umeå Plant Science Centre, Swedish University of Agricultural SciencesUmeå, Sweden

**Keywords:** LRR-RLK, phylogeny, genome duplication, peptide signaling, leaf abscission, fruit abscission, *Populus*, oil palm

## Abstract

The peptide INFLORESCENCE DEFICIENT IN ABSCISSION (IDA), which signals through the leucine-rich repeat receptor-like kinases HAESA (HAE) and HAESA-LIKE2 (HSL2), controls different cell separation events in *Arabidopsis thaliana*. We hypothesize the involvement of this signaling module in abscission processes in other plant species even though they may shed other organs than *A. thaliana*. As the first step toward testing this hypothesis from an evolutionarily perspective we have identified genes encoding putative orthologs of IDA and its receptors by BLAST searches of publically available protein, nucleotide and genome databases for angiosperms. Genes encoding IDA or IDA-LIKE (IDL) peptides and HSL proteins were found in all investigated species, which were selected as to represent each angiosperm order with available genomic sequences. The 12 amino acids representing the bioactive peptide in *A. thaliana* have virtually been unchanged throughout the evolution of the angiosperms; however, the number of *IDL* and *HSL* genes varies between different orders and species. The phylogenetic analyses suggest that *IDA, HSL2*, and the related *HSL1* gene, were present in the species that gave rise to the angiosperms. *HAE* has arisen from *HSL1* after a genome duplication that took place after the monocot—eudicots split. *HSL1* has also independently been duplicated in the monocots, while *HSL2* has been lost in gingers (Zingiberales) and grasses (Poales). *IDA* has been duplicated in eudicots to give rise to functionally divergent IDL peptides. We postulate that the high number of *IDL* homologs present in the core eudicots is a result of multiple whole genome duplications (WGD). We substantiate the involvement of IDA and HAE/HSL2 homologs in abscission by providing gene expression data of different organ separation events from various species.

## Introduction

Plant architecture is dependent on the balance between cell division and cell elongation, proliferation and differentiation, as well as formation and abscission of organs. Plants are sessile organisms that over evolutionary time have adapted to their environment. They have developed reproductive strategies involving mechanisms that in various ways facilitate fruit and seed dispersal. The shedding of organs that have served their purpose is furthermore part of normal development, but detachment can also be a response to injury or environmental changes. The model plant *A. thaliana* shed individual floral organs (petals, sepals, and stamen) shortly after pollination (Bleecker and Patterson, 1997), and displays opening of the valves of the mature siliques (so called dehiscence), and thereafter seed dropping. Other species may shed leaves (e.g., deciduous trees like aspen, *Populus tremula*) and whole flowers (e.g., Citrus), or fruits (e.g., tomato, palms).

Despite variation in the sites of abscission in different species of flowering plants, various abscission events are similar at the cellular level in that they take place in specialized abscission zones (AZs). AZs are either generated already during the development of the organ, or can be induced in response to hormonal or environmental cues (Patterson, 2001; Roberts et al., 2002; Lewis et al., 2006; Aalen, 2011). Organ detachment is a cell separation process that involves dissolution of the middle lamella between adjacent AZ cell files presumably through the action of a number of different cell wall remodeling and degrading proteins, e.g., polygalacturonases, expansins, XTHs, and endoglucanases (Cho and Cosgrove, 2000; González-Carranza et al., 2007; Lashbrook and Cai, 2008; Meir et al., 2010; Liu et al., 2013; Niederhuth et al., 2013; Tsuchiya et al., 2015). One can assume that there is a need for tight regulation and coordination of expression of genes involved in cell separation processes, since cell wall weakening and breakdown could render the plant more susceptible to pathogen attack, and premature organ loss would reduce reproductive success.

In *A. thaliana* a peptide ligand—receptor system responsible for such tight regulation of floral organ abscission has been identified (Butenko et al., 2003; Stenvik et al., 2008). This signaling module consists of the secreted peptide IDA encoded by the gene *INFLORESCENCE DEFICIENT IN ABSCISSION* (At1g68765), and the two leucine-rich repeat (LRR) receptor-like kinases (RLK) HAESA (HAE) and HAESA-LIKE2 (HSL2) (Cho et al., 2008; Stenvik et al., 2008). In both *ida* and *hae hsl2* mutants floral organs are retained indefinitely. *IDA* belongs to a small gene family in *A. thaliana* with five additional *IDA-LIKE* (*AtIDL*) members that to a varying degree can substitute for *IDA* function (Butenko et al., 2003; Stenvik et al., 2008). IDA and the IDL proteins share a short C-terminal sequence encompassing the bioactive peptide (Stenvik et al., 2008), which for IDA has been delineated to a 12 amino acids (aa) long proline-rich peptide (hereafter called mature IDA, mIDA) using genetic and biochemical tools (Stenvik et al., 2008; Butenko et al., 2014). Overexpression either of *IDA* or *AtIDL* genes, results in early abscission and enlarged abscission zones (AZ) at the base of the floral organs, but when expressed using IDA's own promoter, only *IDA* and *AtIDL1* can fully complement the *ida* mutant phenotype. Together this may suggest that AtIDL2-5 peptides have lower affinity to HAE and HSL2, and possibly signal through (a) different, but related receptor(s) (Stenvik et al., 2008). There are more than 600 genes in *A. thaliana* encoding receptor-like kinases, and of these more than 200 encode extracellular domains with LRRs of varying length assumed to bind small peptides (Shiu and Bleecker, 2001). Only about a dozen of these have been matched with a ligand (Butenko et al., 2009). The majority of these, including HAE and HSL2, belong to the RLK subclass XI, which has more than 20 LRRs.

Interestingly, ectopic expression of IDA in *A. thaliana* leads to abscission of fruit, flowers, branches, and cauline leaves at vestigial AZ at the base of these organs, which are sites of cell separation in a number of other species, but normally not in *A. thaliana* (Stenvik et al., 2006). Additionally, the IDA-HAE/HSL2 signaling module is involved in a quite different cell separation process, namely lateral root emergence (Kumpf et al., 2013). IDA and the receptors are expressed in the endodermal, cortical, and epidermal cells overlaying lateral root primordia, induce expression of cell wall remodeling genes and facilitate emergence without cellular disruption (Aalen et al., 2013; Kumpf et al., 2013).

The objective of the presented work has been to investigate to what extent orthologs of *IDA, HAE*, and *HSL2* are present in other angiosperms irrespective of which organs they shed, and whether gene expression data could substantiate a role of these orthologs in cell separation events.

## Materials and methods

### Phylogenetic analyses

Sequences used in this study were obtained from various databases [NCBI-protein, NCBI-Assembly, Phytozome, Ancestral Angiosperm Genome Project (AAGP), Comparative Genomics (CoGe)] using either tBLASTn or BLASTp (as of 3.2015) with an *A. thaliana* IDL or HSL query. Outgroup taxa for both monocot and eudicot receptors were closely related *A. thaliana* representations within the subclass XI LRR-RLKs. IDA was inferred without an outgroup. An aa alignment (~59 aa) of angiosperm IDA without the N-terminal secretion signal and LRR-RLK group (full length 1926 aa) was constructed using MAFFTv6 E- INS-I model under default settings (Katoh and Toh, 2008). The resulting alignments were checked with Gblocks v0.91b (Castresana, 2000), under the least stringent parameters (small final block, gap positions in final block and less strict flanking), to exclude poorly aligned positions and divergent regions from subsequent phylogenetic inferences. Multiple homologous copies of HSL1/HSL2/HAESA (At1g28440/At5g65710/At4g28490) for each species were removed to reduce phylogenetic noise; in all cases the copy with most sequence data and thereafter shortest branch length was retained for further analysis. ProtTest v2.4 (Abascal et al., 2005) determined LG as the optimal evolutionary model. Maximum Likelihood (ML) analyses were performed with RAxML-VI-HPCv8.0.26, using the PROTCATLG model with 25 rate categories (Stamatakis, 2006). The most likely topology was established from 100 separate searches and bootstrap analyses were performed with 500 pseudoreplicates.

All model estimation and phylogenetic analyses were done using either Lifeportal (https://lifeportal.uio.no) or the abel server at the University of Oslo. All alignments constructed as part of this study are available as Supplementary Data sets, and will in addition be made freely available as Supplementary resources through the authors' Research Gate pages (https://www.researchgate.net/profile/Reidunn_Aalen) and (https://www.researchgate.net/profile/Russell_Orr2).

### Plant material

*A. thaliana* has been transformed with *HAE* and *HSL2* promoter::GUS constructs in the vector pMDC162 made using Gateway Cloning Technology (Curtis and Grossniklaus, 2003) with the same promoter region upstream of the coding sequence (1601 bp and 2300 bp, respectively) as previously reported for *HAE* and *HSL2* constructs with YFP reporter (Kumpf et al., 2013).

Fully expanded leaf blades of hybrid aspen, *Populus tremula* L. X *P. tremuloides* Michx.; clone T89, were shaded in aluminum foil to induce abscission and total RNA was extracted from 3 mm-thick leaf axils 6 days after shading started using RNeasy Plant Mini Kit (Qiagen). Two micrograms of total RNA was used as a template for reverse transcription with the QuantiTect Reverse Transcription Kit (Qiagen). Quantitative real-time PCR (qPCR) was performed using SYBR Green I Master in combination with a LC4800 (Roche Diagnostics) qPCR machine. Primers are specified in Supplementary Table S1. Expression was normalized to *PtACTIN1* expression.

mRNA was isolated from AZs of unripe and ripe of oil palm (*Elaeis guineensis*) fruit, and AZs of fruit treated with ethylene as described previously (Roongsattham et al., 2012). Primers were designed to fit the various *EgIDA* genes, *EgHSL2*, and *EgHSL1* (Supplementary Table S1). qPCR was performed as previously described (Roongsattham et al., 2012). All expression was normalized to the *EgEf*α*1* (accession number: AY550990) expression. No change of *EgEf*α*1* transcript accumulation was found in the fruit tissues treated or not treated with ethylene. Controls were conducted to validate the absence of DNA in each sample.

## Results

### HSl2 has been lost in the gingers and the grasses, and HAE is present only in eudicots

The *A. thaliana* HAE and HSL2 receptors are closely related (60% similar and 45 % identical aa) but HAE is even more closely related to HSL1 (73% similarity, 58% identical aa), an RLK so far with unknown function. BLASTp and tBLASTn were used to identify putative HSL1 angiosperm orthologs in addition to orthologs of HAE and HSL2. Other related receptors from the LRR-RLK subclass XI were used as outliers. Protein sequences from 60 species belonging to 24 orders covering basal angiosperms, monocots, basal eudicots, and core eudicot clades were used to determine the phylogenetic relationship.

**Figure 1:**
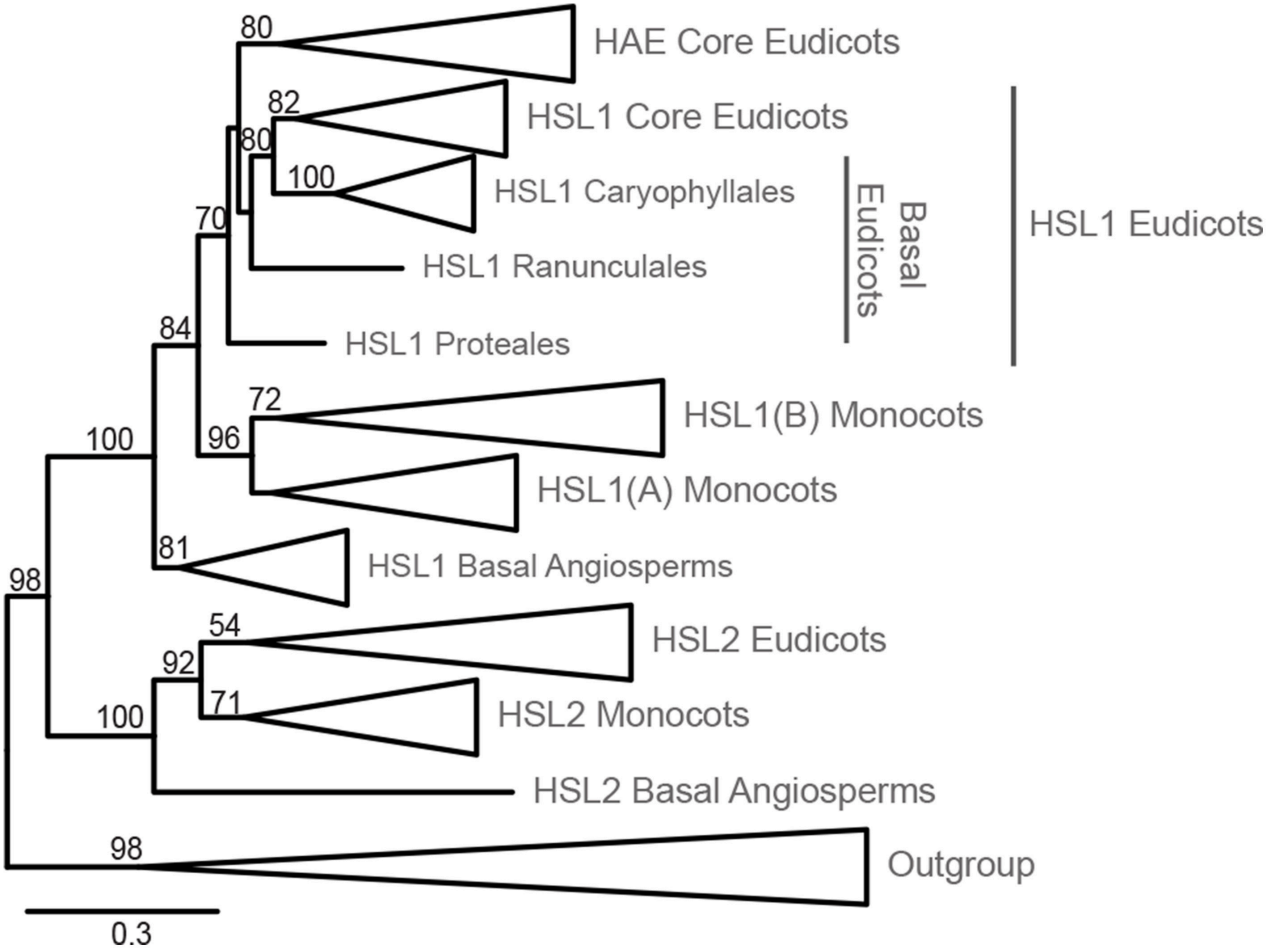
**Phylogeny of HSL LRR-RLK evolution within Angiosperms**. Phylogeny inferred from ML with 145 ingroup taxa and 784 amino acid characters. The phylogeny has been collapsed at different taxonomic levels and shows only bootstrap values >50. The expanded tree is presented in Supplementary Figure S1, which includes an overview of species comprising each order. The alignments used for the construction of this phylogeny are available as Supplementary Data Sheet 1 and 2.

The LRR-RLK ingroup is monophyletic, separated from the outgroup with high support (98% Bootstrap, BP) (Figure 1, Supplementary Figure S1). HSL2 and HSL1/HAE represent two fully supported distinct clades (100% BP). In the HSL2 clade, the basal angiosperm copy is first to diverge within a fully supported grouping, leaving a highly supported (92% BP) monocot/eudicot clade. Subsequently, a moderately supported (71% BP) monocot clade diverges, leaving a poorly supported (54% BP) eudicot monophyly. HSL2 is however missing in the taxa from the orders Poales and Zingiberales (Supplementary Figure S1).

The HSL1/HAE clade is fully supported with the basal angiosperm copy being first to diverge leaving a moderately supported (84% BP) monocot/eudicot clade (Figure 1). The monocot HSL1 copy diverges next forming a highly supported clade (96% BP). Two groupings are found within the monocot clade; the first—constituting all monocot orders (HSL1A)—is unsupported (40% BP), but excluded from the second, longer-branching HSL1B. This duplicate HSL1 clade for the orders Arecales (palms), Poales, and Zingiberales, has moderate support (72% BP) (Figure 1, Supplementary Figure S1). The eudicot HSL1/HAE grouping is moderately supported (70% BP). The HAE clade, representing Vitales in addition to all Rosids and Asterids, is moderately supported (80% BP) and excludes the basal eudicot orders Ranunculales, Proteales, and Caryophyllales which have a closer affinity to the moderately supported (82% BP) eudicot HSL1 grouping. The fully supported Caryophyllales clade forms a moderately supported (80% BP) monophyly with the eudicot HSL1 grouping.

Our analyses revealed that all angiosperms have *HSL1* genes, in in basal angiosperms and some monocots, and almost all eudicots in combination with a *HSL2* gene. Only the core eudicots have both a *HAE* and a *HSL2* gene. In *A. thaliana HAE* and *HSL2* are redundant in function with respect to floral organ abscission, as both genes must be mutated to give the abscission-deficient phenotype (Cho et al., 2008; Stenvik et al., 2008).

### HAE and HSL1 differ from HSL2 especially in their kinase domains

LRR-RLK proteins consist of three domains with different functions: the intracellular kinase domain that confers the signaling output upon ligand binding, the transmembrane domain that anchors the receptors in the plasma membrane, and the extracellular LRR to which the ligand will bind (called the ectodomain). As these three domains have different functions, the selective pressure for rejecting or preserving new mutations might differ.

The intracellular kinase domain has typically two regions: the N-terminal lobe with several β strands and a conserved ATP binding site, and the larger C-terminal part with a number of α-helices, and an activation loop with serine/threonine [Ser(S)/Thr(T)] target residues for phosphorylation. In the BRASSINOSTEROID INSENSITIVE1 (BRI1) LRR-RLK several such residues are phosphorylated (Wang et al., 2005). These residues are well conserved in the *A. thaliana* HAE, HSL1 and HSL2 receptors and their orthologs in other species (Supplementary Figure S2A). This includes the residue corresponding to S861 of HAE, which is subject to autophosphorylation (Taylor et al., 2013), and several residues predicted to be targets of Ser/Thr kinases (e.g., by using PhosPhAt http://phosphat.uni-hohenheim.de/) (Durek et al., 2010). However, in loops between helices positions of putative phosphorylation sites differ in HSL2 receptors compared to HAE/HSL1. Furthermore, HSL2 receptors lack a C-terminal extension with putative phosphorylation sites (Supplementary Figure S2A). The predicted secondary structures of the transmembrane domains of HAE, HSL1, and HSL2 are similar, with a membrane-spanning, hydrophobic α-helix flanked by conserved aa (Supplementary Figure S2B). HSL2 sequences differ from HAE and HSL1 sequences by a shorter α-helix. HAE sequences can be distinguished from HSL1 by a two aa deletion near the end of the transmembrane domain (Supplementary Figure S2B).

**Figure 2:**
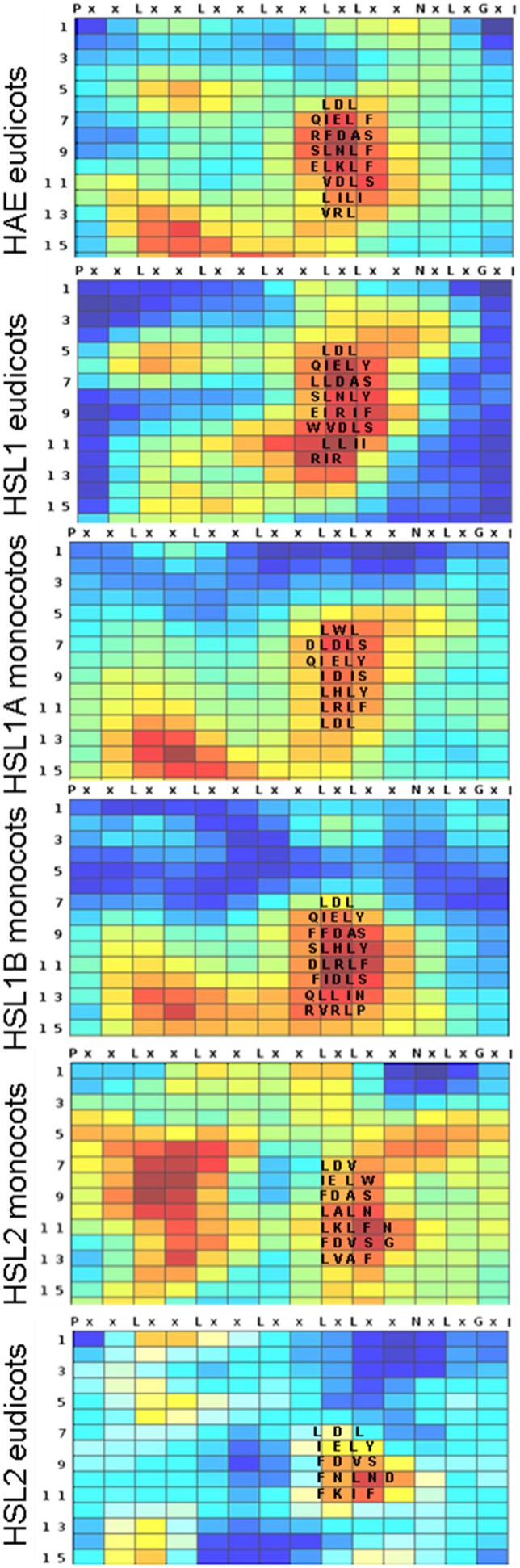
**Conserved amino acids in the LRR of HSL proteins**. Heatmaps generated using Repeat Conservation Mapping (RCM) of LRR domains (http://144.92.198.58/main/main.php) reflecting degree of identity and similarity of aa residues in a given position (X axis) in a given repeat (Y-axis) on the surface of the LRR domain of HAE orthologs from the eudicots, HSL1 from eudicots, HSL1A and B from monocots, HSL2 from eudicot and from monocots. The heatmaps are generated using the same ortholog sequences as used for the phylogenetic analysis (Supplementary Figure S1). The alignments used for heatmap construction are available as Supplementary Data Sheet 2.

So far, the exact location of the *A. thaliana* mIDA peptide when binding HAE or HSL2 is not known, however, in other cases small peptides have been shown to bind consecutive repeats on the inside pocket of the LRR (Sun et al., 2013). Each repeat has typically 24 residues with six evenly distributed conserved leucine residues (Leu, L), which together with asparagine (Asn, N) and glycine (Gly, G) in conserved positions build the structural framework of the ectodomain (Figure 2). These residues are almost invariable and have therefore limited informative value in a phylogenetic perspective. In contrast, conserved non-structural residues are interesting since selection pressure conserving residues involved in ligand binding is likely. To identify such residues we employed the Repeat Conservation Mapping (RCM) (http://144.92.198.58/main/main.php), which relies on a set of algorithms to identify predicted functional sites of LRR domains. This is accomplished by identifying the extent of conservation of different aa patches on the predicted surface of LRR and generating a colored heat map, displaying how conserved an aa is in a given position in a set of orthologs (Helft et al., 2011). For all the HSL receptors a region with highly conserved aa was stretching from the seventh LRR with the common signature sequence QIEL[Y,F], to the fourteenth LRR (Figure 2). The signatures were more similar between the HAE and HSL1, than HSL2.

### The bioactive IDA peptide is conserved in all flowering plants

**Figure 3:**
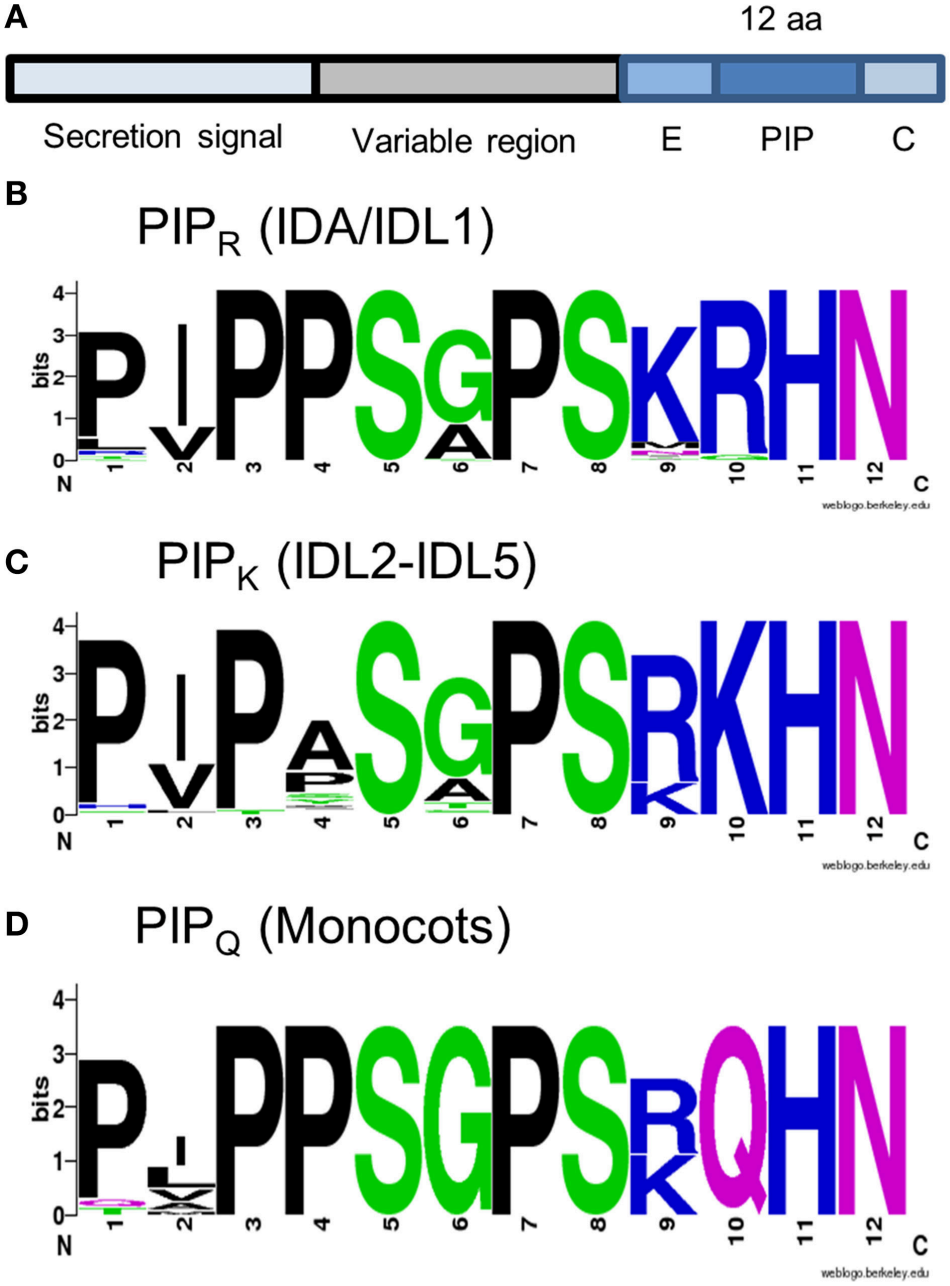
**IDA and IDL peptides**. **(A)** Structure of IDA and IDL prepropeptides. **(B–D)** Peptide consensus sequences as indicated. The alignment used for the construction of the peptide logos is available as Supplementary Data Sheet 3.

IDA and IDL are secreted peptides generated from prepropeptides (Figure 3A), where the hydrophobic N-terminus is a signal directing the protein to the secretory pathway. The variable middle part and the C-terminus are assumed to be cleaved off in the apoplastic space to release a mature 12 aa peptide named PIP after the three first residues, PIPPSAPSKRHN (Butenko et al., 2003). Synthetic mIDA peptide with hydroxylation on the central proline (Pro, P) can bind and activate HSL2 efficiently, and HAE at a higher concentration (Butenko et al., 2014). The Pro residues in positions 2, 3, and 7, serine (Ser, S) in positions 5 and 10, and histidine (His, H) asparagine (Asn, N) at the end are found in all *A. thaliana* IDA/IDL peptide sequences. Experiments where the part of the *AtIDA* gene encoding PIP was swapped with the corresponding *AtIDL* sequence and the recombinant *AtIDA-IDL* gene introduced in the *ida* mutant, indicate the importance of different aa residues (Stenvik et al., 2008). The AtIDL1 peptide (LVPPSGPSMRHN) complements the mutation, suggesting that the initial Pro is of little importance, that the hydrophobic isoleucine (Ile, I) and the small central Ala residues can be exchanged with the hydrophobic valine (Val, V) and the small Gly, respectively, without affecting the biological activity, and furthermore that a positively charged aa in position 9 is no absolute requirement. In contrast, *AtIDL2, AtIDL3, AtIDL4*, and *AtIDL5* cannot fully complement the *ida* mutation (Stenvik et al., 2008). Their PIP motifs are characterized by arginine-lysine (ArgLys, RK) in position 9 and 10, in contrast to the LysArg (KR) found in mIDA.

BLAST and tBLASTn searches were executed using the C-terminal sequences of *A. thaliana* IDA and IDL (i.e., PIP extended with eight aa N-terminally, and the C-terminal aa, Figure 3A) BLAST hits were curated for length of the coding region (<120 aa), presence of hydrophobic secretion signal, the C-terminal position of the conserved PIP motif, and the presence of the conserved Pro, Ser, and HisAsn residues. IDA and/or IDL sequences were identified in all groups in our data sets.

We were unable to infer a phylogeny over the evolution of IDA, due to the short sequence of the conserved peptide. IDA lacks phylogenetic information and is also present in a high number of copies in many species; any possible pattern was unclear, with no clear clades, branching patterns, and a general lack of stability. Despite this we attempted an inference based on multiple datasets (PIP, EPIP, and EPIP-C, Figure 3A), considering species that have undergone fewer genome duplications (Vanneste et al., 2014), however, without success.

As an alternative approach to understand the evolution of IDA/IDL peptides, we grouped the different PIP motifs of the BLAST-identified proteins based on their amino acid composition. In all eudicots, peptides could be classified either as IDA/IDL1 ([P,L][V,I]PPS[A,G]PSK**R**HN) or like IDL2-5 (PIP[A,T,H,P]S[A,G]PSR**K**HN), named PIP_R_ and PIP_K_ respectively based on the residue in position 10 (Figures 3B,C). Both motifs were found in every eudicot species tested. In the monocot dataset PIP_R_ was found in the orders Arecales and Zingiberales. Additionally, we identified a version in all monocots with close resemblance to PIP_R_, but containing a glutamine (Gln, Q) in position 10 (PIP_Q_, Figure 3D).

### The *IDL* gene family has expanded in many taxa of core eudicots

About 65 million years (Myr) ago after the split of the core eudicots into Rosids and Asterids, whole genome duplication (WGD) took place in a number of eudicot lineages and expanded both the number of species and the gene number of each species (Van de Peer et al., 2009). In line with this, detailed inspection suggests independent evolution especially of the *IDL2-5* genes in these two clades. Soybean (*Glycine max*), common bean (*Phaseolus vulgaris*) and *A. thaliana* belong to the Rosids, the beans representing the families of legumes (*Fabales*), and *A. thaliana* belonging to the *Brassicales*. Soybean has six pairs of *IDL* genes, each pair represented only once in common bean, consistent with a late genome duplication in soybean after the separation from bean (Tucker and Yang, 2012). Tomato (*Solanum lycopersicum*), potato (*Solanum tuberosum*), and tobacco (*Nicotiana* ssp.) belong to the family *Solanales* in the Asterid clade, and are considered diploid species. However, analyses of inter- and intrachromosomal duplications in the tomato and potato genomes suggest a second more recent WGD in the common ancestor of *Petunoideae, Nicotianoideae*, and *Solanoideae* (Song et al., 2012). We have found pairs of almost identical *IDL* genes both in *N. benthamiana* and tomato that are more similar within than between the species (Supplementary Figure S3A), suggesting that additional independent duplications have taken place after the divergence of *Nicotianoideae* and *Solanoideae*. Similarly, we have previously suggested that the *AtIDL2* and *AtIDL3* genes in *A. thaliana* result from a recent duplication event (Stenvik et al., 2008).

### IDA ligands and HSL receptors are expressed in a variety of AZs and species

Having identified orthologs of IDA and its receptors in a variety of species, we examined in the literature and experimentally whether IDA/IDL1 and the relevant receptors were likely to be involved in cell separation events.

#### Floral organ and flower abscission

Abscission plays an important role during plant reproduction. It is common among eudicots (including *A. thaliana*) to abscise turgid floral organs after pollination, when they no longer are functional (van Doorn, 2001). In some species the function of organs change during development, exemplified by tepals in *Eriophyllum* spp. (Asterales) and *Polygonum* spp. (Caryophyllales) that first act as protection of reproductive organs and later as organs involved in seed dispersal. In monocots floral organs may wither without abscission (van Doorn, 2001, 2002), or the perianth is retained and will cover the fruits during seed maturation. Hence, in such settings IDA and its receptors are not expected to be active. We postulate that the signaling system is intact in monocots in species where abscission of individual organs takes place when the tepals are free, like in tulips (*Tulipa* spp., Liliales). Unfortunately genomic and transcriptomic data are scarce for many monocots, especially the Liliales which have gigantic genomes (Shahin et al., 2012).

**Figure 4:**
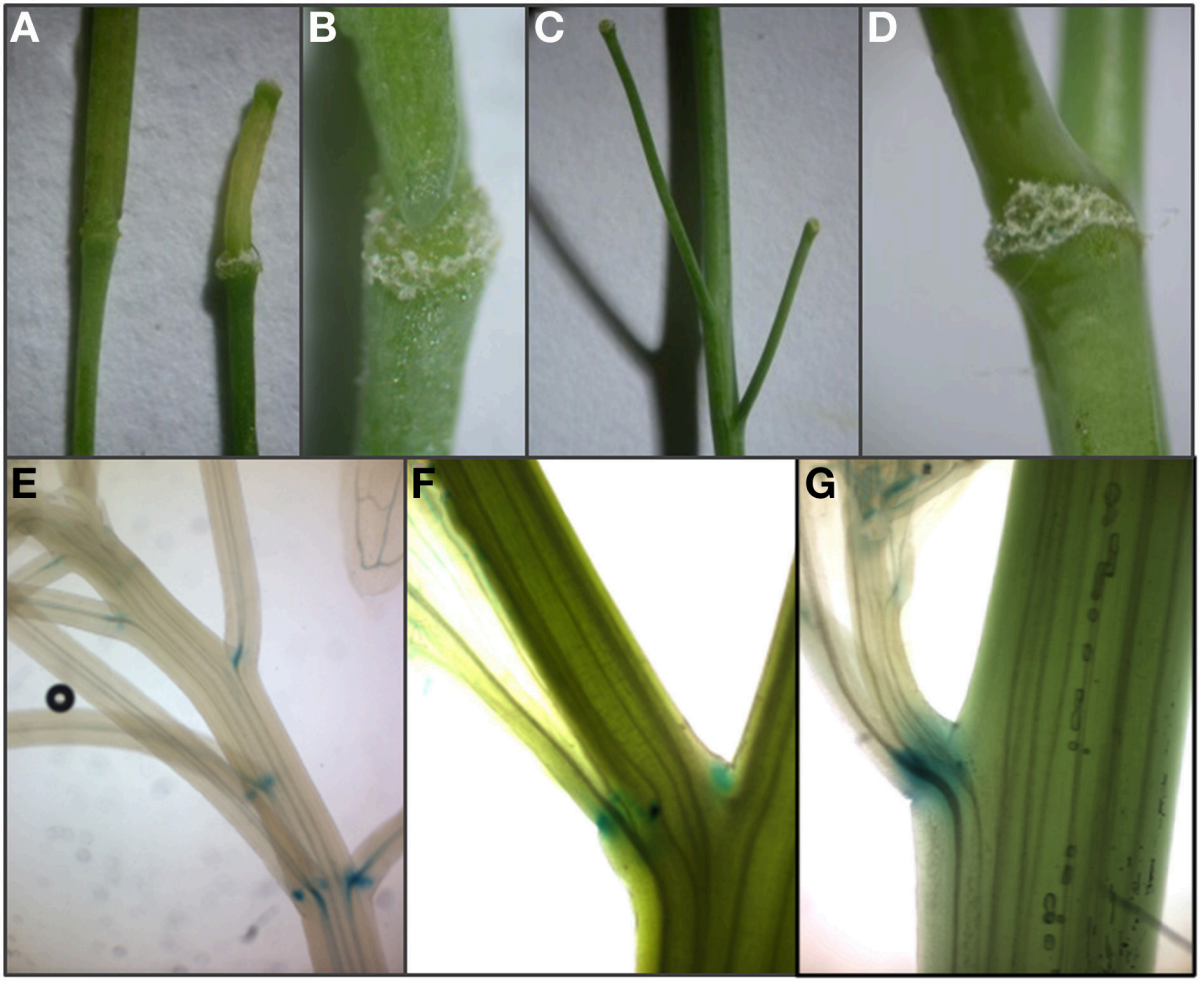
**Expression of *HAE* and *HSL2* promoter: GUS constructs at sites of ectopic abscission**. **(A–C)** Enlargement of AZ and premature abscission of whole flowers and immature fruits compared to wild type silique (to the left in **A**) in *A. thaliana* plants overexpressing AtIDL1. **(D)** Enlarged vestigial AZ after abscission of a cauline leaf in *A. thaliana* overexpressing AtIDL1. **(E,F)** *pHAE:GUS* expression and **(G)** *pHSL2:GUS* expression in vestigial AZs at the bases of pedicels, branches, and cauline leaves.

Normally *A. thaliana* does not shed cauline leaves, whole flowers or fruits. However, ectopic expression of IDA and IDL peptides in *A. thaliana* leads to induction of abscission in a HAE/HSL2-dependent manner at the base of pedicels, cauline leaves, and inflorescence branches, rather than a general separation of cells in the plant (Figures 4A–D) (Stenvik et al., 2006). These are sites of abscission in other species. Interestingly, *A. thaliana* lines transformed with promoter:GUS constructs for *HAE* or *HSL2* show that the receptor genes are expressed in the vestigial AZs at these sites (Figures 4E–G).

**Figure 5:**
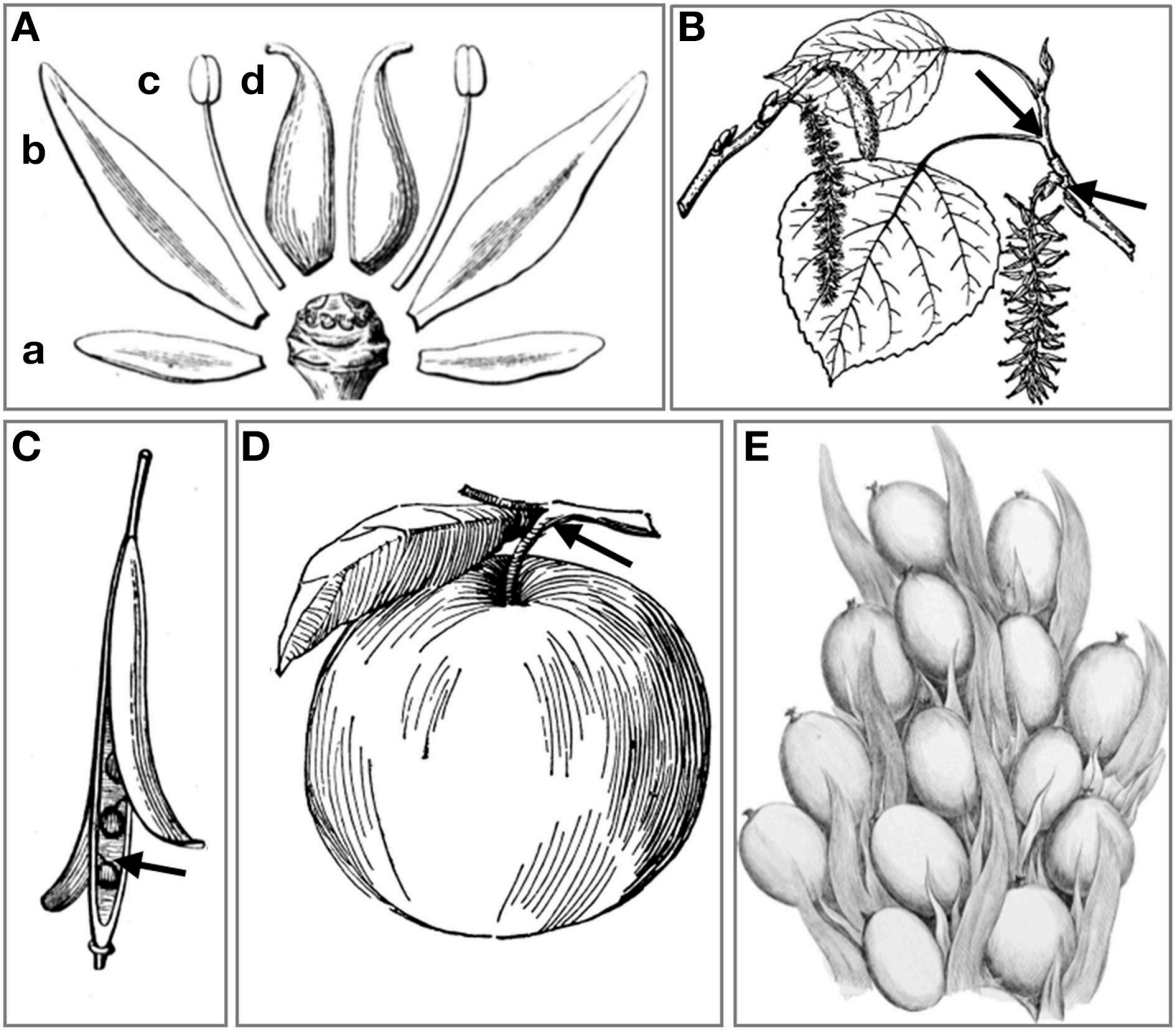
**Modes of abscission**. **(A)** Abscission of (a) sepals, (b) petals, (c) stamen, and (d) carpels. **(B)** Abscission of leaves at the axil of the pedicel, and abscission of entire male inflorescence (catkin) in *Populus* spp. **(C)** Opening of valves in dehiscence zones of dry many-seeded capsules, and abscission of individual seeds. **(D)** Abscission of fleshy fruits at AZ on pedicel. **(E)** The oil palm drupe fruit are tightly arranged within spikelets and abscise one by one when ripe. **(A–D)** Image courtesy the private collection of Roy Winkelman. First published in Gray (1858) and Foster (1921). **(E)** Image courtesy Missouri Botanical Garden. http://www.botanicus.org.

Fall of whole flowers is less common than floral organ abscission (Figure 5A), but occurs in both monocot and eudicot species, when pollination or fertilization fail (van Doorn, 2002). Abscission of immature fruit is a normal event in several cultivated species. In *Citrus* spp. (Sapindales), abscission of flowers and young fruit results from cell separation at an AZ at the base of the floral pedicel. The *CicIDA3* gene, expressed in *Citrus clementina* AZs, encodes a protein with a PIP motif identical to mIDA with the exception of a Gly instead of an Ala in position 6 (Estornell et al., submitted). Overexpression of CicIDA3 in *A. thaliana* induced the same phenotypic changes as have been shown for overexpression of IDA and AtIDL1 (Figures 4A–D) (Estornell et al., submitted), suggesting that the citrus prepropeptide undergo the correct processing, that the *Citrus* peptide can activate IDA's receptors and thus provides experimental evidence supporting a function in citrus abscission events.

#### Leaf abscission

AZs, with layers of rather undifferentiated small cells, are normally formed during organ development both in *A. thaliana* and other species. Additionally, ethylene may promote the formation of new AZs and thereby induce abscission (Roberts et al., 2000). A thorough investigation of IDA and HAE/HSL2 in connection with ethylene-induced leaf abscission has been performed in soybean (*Glycine max*), common bean and tomato (Tucker and Yang, 2012). Of the five genes encoding tomato IDA/IDL peptides, one with a PIP motif identical to that of mIDA was expressed in ethylene-induced AZs on leaf pedicels. Likewise, the GmIDA2a and 2b, which had the highest expression level in leaf AZ and the highest relative expression ratio between AZ and petiole tissue both in the presence and absence of ethylene, are the paralogs most similar to AtIDA, which also display ethylene independent expression. The GmHAE3b and GmHAE5a/5b with the highest similarities to *A. thaliana* HAE and HSL2, respectively, were also adequately expressed in the petiole (Tucker and Yang, 2013). Thus, these expression patterns support the involvement of the IDA-HAE/HSL2 module in leaf abscission both in Rosids and Asterids.

One of the most visible of all shedding processes in angiosperms is the leaf fall (Figure 5B) at the beginning of the dormant (i.e., cold or dry) season in deciduous trees, triggered by seasonal reduction of the photoperiod and temperature (Taylor and Whitelaw, 2001; Keskitalo et al., 2005). Autumnal leaf abscission provides deciduous trees with resistance against drought and freezing damage (Fischer and Polle, 2010; Zanne et al., 2014). Species with N-fixing symbionts like alder (*Alnus* spp., Fagales) can afford to lose green leaves, while other deciduous trees, like *Populus* ssp. (Malpighiales), withdraw elements of valuable molecules (as chlorophyll) and shed yellowish or reddish leaves (Keskitalo et al., 2005; Fracheboud et al., 2009). Hence, the senescence process that allows trees to conserve resources and prepare for a dormant period terminates in a cell separation event in preformed AZs at the base of the pedicels.

**Figure 6:**
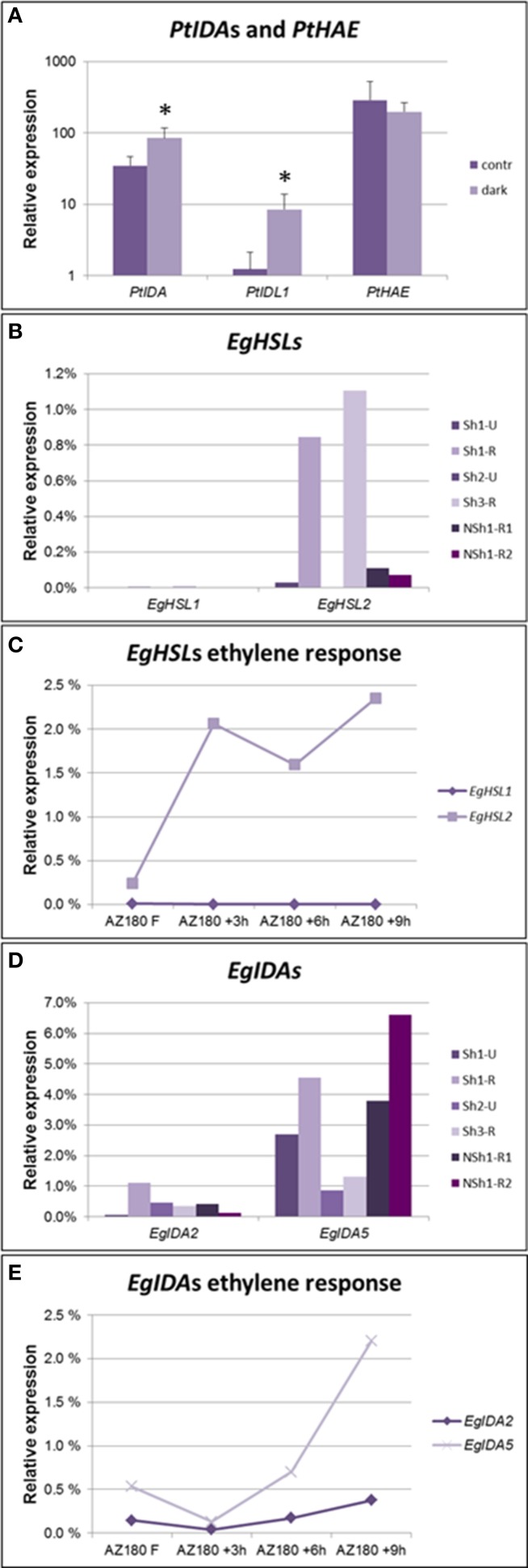
**Expression levels of genes encoding IDA ligands and HSL receptors in AZs**. **(A)** qPCR detecting increased expression levels of *Populus IDA* genes, but not the *PtHAE* gene in axils of shade-treated leaves prone to abscise compared to axils in non-shaded, non-abscission aspen leaves. qPCR, averages and standard deviations of three biological replicates. Normalized to *PtACTIN1* expression. ^*^p < 0.05, *t*-test, non-shaded vs. shaded. **(B)** qPCR analysis of oil palm *EgHSL1* and *EgHSL2* expression in AZs from unripe (Sh1-U and Sh2-U) not actively abscising fruit and AZs of ripe (Sh1-R, Sh3-R) actively abscising fruit, as well as AZs from ripe fruit of a non-abscising tree (NSh1-R1, NSh1-R2). **(C)** qPCR analyses of oil palm *EgHSL* expression during ethylene-induced abscission in ripe 180 DAP fruits. Samples were taken after 0, 3, 6, and 9 h treatment with ethylene. Similar results were obtained when treating 145 DAP fruits (Supplementary Figure S3C). In both experiments fruit separated by 9 h of ethylene treatment. **(D)** qPCR analysis of oil palm *EgIDA2* and *EgIDA5* expression in the AZs of unripe (Sh1-U and Sh2-U) not actively abscising fruit and AZs of ripe (Sh1-R, Sh3-R) actively abscising fruit, as well as AZs from ripe fruit of a non-abscising tree (NSh1-R1, NSh1-R2). Expression levels of additional *EgIDA* genes are found in Supplementary Figure S3D. **(E)** qPCR analyses of oil palm *EgIDA2* and *EgIDA5* expression during ethylene-induced abscission in ripe 180 DAP fruits. Samples were taken after 0, 3, 6, and 9 h treatment with ethylene. Similar results were obtained when treating 145 DAP fruits (Supplementary Figure S3E). In both experiments fruit separated by 9 h of ethylene treatment.

Leaf abscission can be induced in hybrid aspen (*Populus tremula* X *P. tremuloides*) by depriving the leaves of light (Jin et al., 2015). In our experimental setup, 50% of the shaded leaves separated from the branch at the axil after 8 days, while non-shaded leaves were not shed. We designed gene-specific primers against the *IDA, IDL1*, and *HAE* orthologs of *Populus* ssp., which seem to have lost the *HSL2* ortholog. The two genes most similar to *AtIDA* were significantly upregulated in the axil upon induction of leaf abscission (Figure 6A). As in soybean, the expression levels of the receptors did not change in the course of this experiment, suggesting that the timing of abscission is rather dependent on the induction of IDA peptides than on the transcriptional regulation of the receptor.

#### Seed and fruit abscission

The fertilized ovule of Angiosperms (the seed) and surrounding maternal tissue that together constitutes the fruit, needs to be separated from the mother plant in one way or another when ripe. In botanical terms there are roughly four main types of fruits: *berries* (fleshy most often many-seeded fruits), *drupes* (fleshy most often one-seeded fruits with hard exocarp), *capsules*, including siliques and pods (dry many-seeded fruits), and *nuts*, including caryopses of grasses (dry one-seeded fruits). Nuts are shed and abscised at the base of the carpels, while many-seeded capsules or pods open along dehiscence zones (Figure 5C). This is seen in a wide range of angiosperms such as orchids (Asparagales), poppies (*Papaver* spp., Ranunculales), and tobacco (*Nicotiana tabacum*, Solanales) as well as *A. thaliana*, before each single seed abscise where the funiculus is attached to the seed. We have earlier reported that *AtIDA* and HAE are expressed in *A. thaliana* dehiscence zones (Butenko et al., 2006).

For fleshy fruit eaten directly from the plant by birds or animals e.g., blueberries (*Vaccinium myrtillus*, Ericales), abscission is not necessitated for seed dispersal. Nevertheless, both larger fruits like apples (*Malus* domestica, Rosales) (Figure 5D) and drupes like olives (*Olea europaea*, Asterides) (Gil-Amado and Gomez-Jimenez, 2013) may abscise at the apex or at the base of the pedicel (fruit stalk), so that animals may pick up the fruits from the ground. In line with this, *HAE* was recently shown to be strongly upregulated in abscising olives compared to the preabscising AZs (Gil-Amado and Gomez-Jimenez, 2013).

To test whether IDA-HAE/HSL2 is involved in control of fruit abscission also in monocots we chose to investigate abscission of the fruits of oil palm (*Elaeis guineensis*, Arecales). The flowers of the oil palm grow in large clusters giving rise to plumb-sized reddish fruits in large bunches (Figure 5E) that have large, preformed, multilayered AZs at the boundary of the mesocarp and the pedicel. Under natural conditions it takes about 160 days from pollination until the fruits are ripe and start separating from the bunches on the trees. mRNA was isolated from AZs of unripe and ripe fruit of trees that abscise their fruit (Shedding—Sh1, Sh2, and Sh3), and additionally from AZs of an unusual tree (Non-shedding, NSh1) that fails to abscise its fruit. We identified genes encoding HSL1 and HSL2 receptors; *EgHSL1* had a very low expression level that did not change over time (Figure 6B). *EgHSL2* on the other hand was low in unripe AZs of shedding trees, as well as AZs of ripe NSh1 fruit, but increased strongly in AZs of ripe fruit of abscising trees. From about 145 DAP, ethylene treatment induce fruit abscission after 9 h. This treatment also induces increased *EgHSL2* expression (Figures 6C, Supplementary Figure S3C). Thus, *EgHSL2* expression was consistently associated with active fruit abscission.

We identified 10 *IDA* oil palm genes that come in five pairs encoding almost identical preproproteins (Supplementary Figure S3B), suggesting recent genome duplication. Expression of five of these genes (*EgIDA2* to *EgIDA6*) was detected in AZs of *E. guineensis* fruits using RT qPCR (Supplementary Figure S3D). In trees showing fruit abscission *EgIDA2, EgIDA4* and the most abundant, *EgIDA5*, all had their highest expression levels when fruit are separating (Figure 6D), and *EgIDA2* and *EgIDA5* could also be induced by ethylene (Figure 6E). These genes encode PIPs near to identical with mIDA; like CicIDA3, EgIDA5 differs only by an exchange of the small Ala with the small Gly in position 6. Interestingly, the *EgIDA* genes were not strictly associated with separation; as transcripts were present also in ripe AZs of the non-shedding tree (Figure 6C). Hence, *EgIDA* are expressed and induced in NSh1 although the receptor gene *EgHSL2* has a very low level of expression.

## Discussion

**Figure 7:**
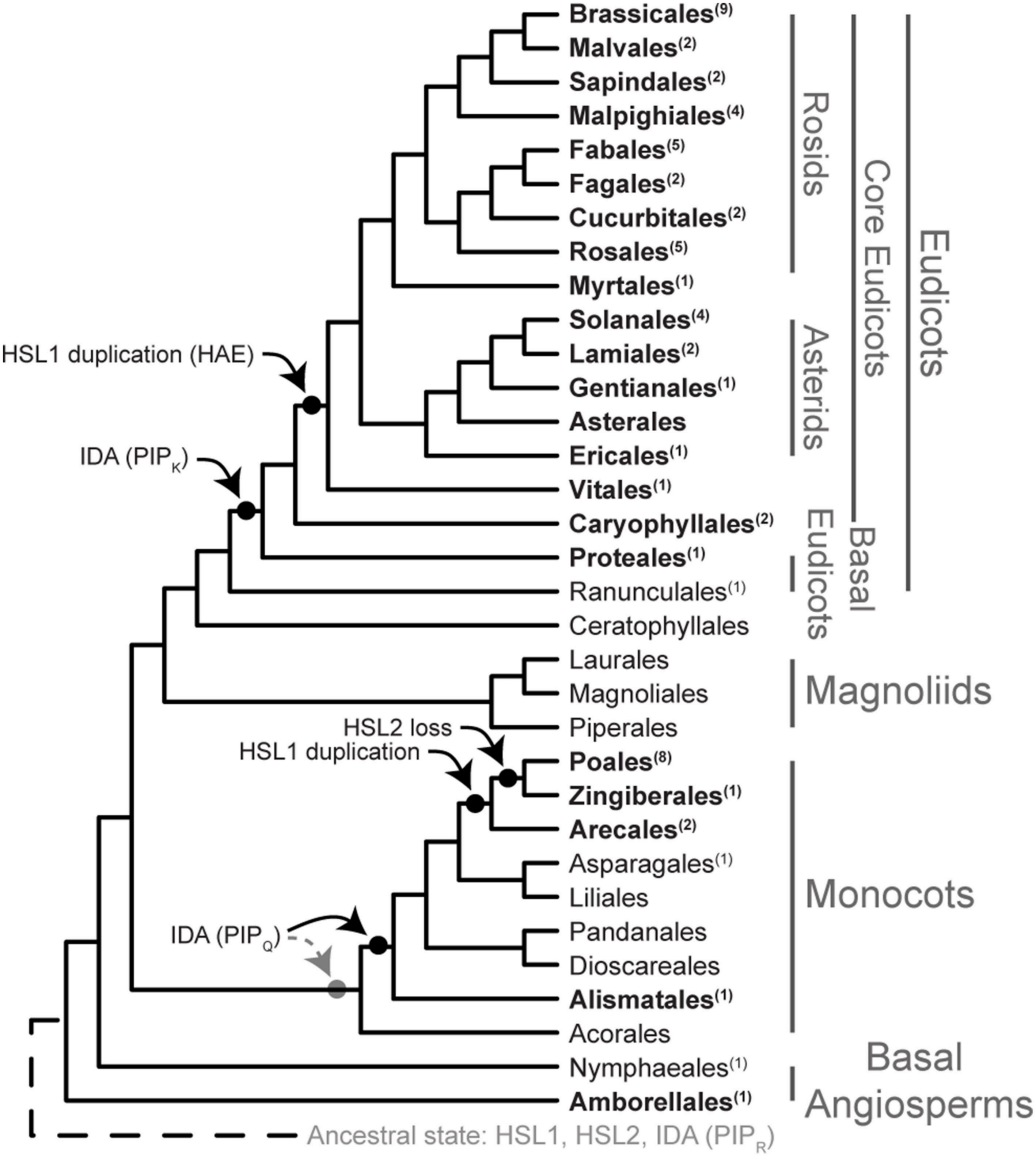
**Evolution of the IDA HAE/HSL2 signaling module**. Phylogeny of Angiosperms adapted from Zhang et al. (2012), Vanneste et al. (2014), Zeng et al. (2014), and Dohm et al. (2014). Taxonomical levels are taken from Zeng et al. (2014). Numbers in superscript behind order names represent the number of species used in the analysis. Order names in bold represent those orders with at least one completely sequenced genome. The two possible evolutionary origins of IDA (PIP_Q_) in monocots are illustrated.

Our phylogenetic analyses of the HSL LRR-RLKs have revealed that the evolution of this receptor family is congruent with that of angiosperm species evolution, and makes it likely that the common ancestor of angiosperms had both a *HSL1* and a *HSL2* gene (Figure 7). Both receptors are present already in the basal angiosperm (e.g., *Amborella*), where stamen abscission is a common feature that facilitates the access of pollinators (beetles and flies bringing pollen from other flowers) to the female organ (Endress, 2010). The *IDA* gene with PIP_R_ motif identified in *Amborella*, may play a role here, similar to IDA's role as a signaling ligand in sepal, petal, and stamen abscission in *A. thaliana* (Butenko et al., 2003, 2014).

While the receptors follow the general evolution of the angiosperms, the PIP_R_ motif likely to represent the peptide released from the propeptide upon secretion, has virtually been preserved without substantial changes throughout the evolution of flowering plants. In a short peptide that presumably shall bind to given aa side chains in the receptor, there are limited possibilities for aa changes that retain the relevant binding properties: Exchange of one small aa for another might pass, but exchange of an aa with different size or charge of the side chain (like a Lys or a Asn for an Arg), may affect the structure of the ligand and its binding properties (Czyzewicz et al., 2015).

The genome of a common ancestor of flowering plants has been suggested to harbor between 10,000 and 14,000 genes (Tang et al., 2008) while a genome size of 25,000–50,000 genes is common in modern angiosperms. This increase is due to a number of WGD, including a triplication in dicots before the separation of the *Vitis*. Two additional duplications and a subsequent extensive loss of duplicated genes occurred in *A. thaliana* (Tang et al., 2008; Van de Peer et al., 2009). Relevant for our analyses is that *HSL1* has been duplicated in two separate events: Firstly, before the split of Vitales, Asterids, and Rosids, within eudicots to form *HAE*, and secondly, within monocots to form a more recent duplication before the split of Arecales. Still, multiple synteny investigations between five different and partly distantly related species (*Arabidopsis, Carica, Populus, Vitis*, and *Oryza*) showed that a total of 61% of the *A. thaliana* genes had preserved their ancestral locations (Tang et al., 2008). There are controversies regarding the placement of the smaller clades of Ceratophyllales and Caryophyllales, as well as Vitales, in relation to the eudicots (Zeng et al., 2014). Our analyses of the three receptor genes support Vitales and Caryophyllales as sister groups of the core eudicots. *HSL1/HAE* duplication is found on syntenic genome regions in the other eudicots, but not rice, supporting that *HAE* was generated by a WGD event (Tang et al., 2008). Specific genomic locations of *AtIDA* and *HSL2* and their neighboring genes in *A. thaliana* are preserved in *Populus* and *Vitis*, respectively (Woodhouse et al., 2011). The *IDL4* is positioned in synteny with other eudicots, but the other *IDL* genes may rather be a result of duplications known to have taken place after the divergence of Brassicaceae and Caricaceae families (Tang et al., 2008).

Duplications provide the opportunity for the new copy to evolve, like giving rise to the PIP_K_ or PIP_Q_ motif varieties. Transition from Arg to Lys or Gln may only require one nucleotide change. Based on available sequence data we hypothesize that IDL4 with PIP_K_ evolved before the split of Proteales, however this variant may have a more basal history within angiosperm evolution. Likewise, the PIP_Q_ version has presumably developed after genome duplication in the monocot lineage, and we hypothesize that PIP_Q_ is present in all monocot orders (Figure 7). Sequence data for Acorales is needed to substantiate this. PIP_R_ may represent the original peptide gene that has been lost from some of the monocot orders: Alismatales and Poales are devoid of the gene, whilst it is present in Arecales and Zingiberales, suggesting independent loss for some lineages. This is more parsimonious than a basal loss within the monocots followed by multiple gains. A final conclusion must await genomic sequencing of species from orders where data at present are lacking.

We have evidence from *A. thaliana* that *IDA* can be substituted with *AtIDL1*, but the other *AtIDL* genes only to a limited extent can substitute for *IDA* function, indicating that their encoded peptides do not interact properly with the HAE and HSL2 receptors (Stenvik et al., 2008). The high similarity between conserved aa in the ectodomain as well as the kinase domain of HSL1 compared to HAE, might suggest HSL1 to be their native receptor. This would require closely related expression patterns for *HSL1* and *IDL* genes. However, at least in Arabidopsis, the expression patterns of *HSL1* and these *IDL* genes do not suggest involvement in floral organ abscission (Cho et al., 2008; Stenvik et al., 2008), and mutant phenotypes are so far lacking. Different methods have lately been suggested for testing of peptide-ligand receptor interactions (Butenko et al., 2009, 2012, 2014). Interaction of the mIDA peptide with HSL2 expressed in *N. benthamiana*, results in an immediate oxidative burst, similar to elicited LRR-RLKs involved in pathogen defense (Butenko et al., 2014). A thousand times more peptide is needed to provoke such a reaction from the HAE receptor under the same conditions, although the two receptors in genetic terms are functionally redundant with respect to control of floral organ abscission (Cho et al., 2008; Butenko et al., 2014). We have found distinct evolutionarily conserved differences between the kinase domains of HAE/HSL1 and that of HSL2. This may explain the differences in readout between HAE and HSL2 in the presence of IDA or IDL peptides (Butenko et al., 2014).

Interestingly, within monocots *HSL1* has been duplicated before the split-off of the Arecales, and *HSL2* has been lost after the split of Arecales, leaving the orders Poales, and Zingiberales devoid of the gene. Abscission still takes place in these orders, e.g., “finger drop” in banana (*Musa* spp.) and caryopsis shedding (diminished in the domesticated species) in the grasses. We have demonstrated that oil palm of the Arecales use *HSL2* and *IDA* genes encoding PIP_R_ peptides in connection to fruit abscission. It is tempting to suggest that the PIP_Q_ version of the IDA peptide have developed to fit one or the other HSL1 receptors.

We have presented examples of the presence of IDA and its receptors in all the major groups of Angiosperms during abscission of floral organs, flowers, immature, and mature fruits and of leaves. The first Angiosperm developed more than 150 Myr ago, and IDA ligands may have been conserved most of this time. At the same time the number of IDA and receptor genes has increased and a variety of modes of organ detachment has developed. This lends support to the notion that it is not what you do, but where and when you do it that is important (Carroll, 2008). New gene copies have presumably made it possible to change place and time for cell separation events.

## Author contributions

IS, RO, KF, XJ, JK, and IN performed the research; IS, RO, UF, TT, and RA analyzed the data; IN and RA designed the research, RA wrote the article with contributions from IS, RO, UF, TT, and IN.

## Funding

This research was supported by grant no 213785 from the Research Council of Norway to RA; by Deutsche Forschungsgemeinschaft (DFG, Fi1661/1-1) to UF; to XJ by Bio4Energy, the Berzelii Centre (Vinnova) and Stiftelsen Mauritz Carlgrens Fond; and by Franco-Thai and Thailand Graduate Institute of Science and Technology (TGIST) scholarships to KF, and funding from PalmElit SAS (KF, TT).

### Conflict of interest statement

The authors declare that the research was conducted in the absence of any commercial or financial relationships that could be construed as a potential conflict of interest.
