# Supplementary material for: Conservation of the abscission signaling peptide IDA during Angiosperm evolution: withstanding genome duplications and gain and loss of the receptors HAE/HSL2
Source: Front Plant Sci. 2015 Oct 30;6:931. doi: 10.3389/fpls.2015.00931 (PMC4627355; doi:10.3389/fpls.2015.00931)
Supplement: Supplementary file 1 [file Presentation1.PDF]

frontiers

Supplementary Material

Conservation of the abscission signaling peptide IDA during Angiosperm evolution: withstanding genome duplications and gain and loss of the receptors HAE/HSL2

Ida M. Stø[1*], Russell J.S. Orr[1*], Kim Fooyontphanich[2], Xu Jin[3], Jonfinn M.B. Knutsen[1], Urs Fischer[3], Timothy J. Tranbarger[2], Inger Nordal[1] and Reidunn B. Aalen[1]

Correspondance: Reidunn B. Aalen: reidunn.aalen@ibv.uio.no

Supplementary Figures

Supplementary Figure S1. Phylogeny of HSL LRR-RLK evolution within angiosperms
Phylogeny inferred from ML with 788 amino acid characters. A collapsed version of the tree is presented in Figure 1.

Supplementary Figure S2. HAE, HSL1 and HSL2 kinase and transmembrane domains.
(A) Aligned amino acid sequences for the kinase domains of the *A. thaliana* proteins HAE, HSL1 and HSL2, and orthologues from basal eudicot (Nn, *Nelumba nucifera*)), and the monocot oil palm (Eg). and for comparison the BRI1 for which the structure has been solved and *in vivo* and *in vitro* phosphorylation sites have been identified (residues inn green)) (Wang et al.Plant Cell 17: 1685-1703, 2005). α-helices and β−strands predicted using I-TASSER (http://zhanglab.ccmb.med.umich.edu/I-TASSER/) are indicated by barrels and arrows, respectively and phosphorylation sites predicted by PhosPhAt 4.0 (http://phosphat.uni-hohenheim.de/) in all of the aligned sequences (in red), in HAE and HSL1 proteins (in blue), or in HSL2 proteins (in yellow). The red line indicates the activation loop of the kinase domain.The yellow barrel is specific for HSL2.
(B) Amino acid sequences for the transmembrane domain of the *A. thaliana* proteins HAE, HSL1 and HSL2, with secondary structure predictions and consensus sequences for angiosperms (4 – Arg or Lys; 6 – Val or Ile). The red rectangle marks the two signature aa present in all HSL1 sequences and deleted in all HAE sequences.

Supplementary Figure S3. Alignment of peptide sequences
(A) Aligned amino acid sequences for IDA and IDLs in selected species.
(B) IDA oil palm sequences aligned with *A. thaliana* sequences.
(C) qPCR analyses of oil palm *EgHSL* expression during ethylene-induced abscission in ripe 145 DAP fruits. Samples were taken after 0, 3, 6 and 9 h treatment with ethylene. Fruit separated by 9h of ethylene treatment.
(D) Expression levels of oil palm *EgIDA1-EgIDA6* expression in the AZs of unripe (Sh1-U and Sh2-U) not actively abscising fruit and AZs of ripe (Sh1-R, Sh3-R) actively abscising fruit, as well as AZs from ripe fruit of a non-abscising tree (NSh1-R1, NSh1-R2).
(E) qPCR analyses of oil palm *EgIDA2* and *EgIDA5* expression during ethylene-induced abscission in ripe 145 DAP fruits. Samples were taken after 0, 3, 6 and 9 h treatment with ethylene. Fruit separated by 9h of ethylene treatment.

Supplementary Data Sheets

Supplementary Data Sheet 1. HSL_LRR-RLK_Align.fasta

Supplementary Data Sheet 2. HSL_LRR-RLK_Masked_Align.fasta

Supplementary Data Sheet 3. IDAepipc.fasta

Supplementary Table S1. Primers

Primer	Sequence 5’ – 3’
EgIDA1_S	AGAGGAGGAATTCGGGCTAC
EgIDA1_AS	ATTTCCGATGGCTTCAGCTC
EgIDA2_S	ACTCCCGGCATTCAACTTC
EgIDA2_AS	TGATGATCGATCGAAAGCTG
EgIDA3_S	GGAGGAATTCGGGCTTCTAC
EgIDA3_AS	AAAGAACCCAACCATGTACATTAAC
EgIDA4_S	AAGAGGCATAACTCGGTGCTC
EgIDA4_AS	GGCAAAAATGATTCAAATAAAGG
EgIDA5_S	GAACGCCTTCACACATTCAG
EgIDA5_AS	TGATCGAAATCCGCATGG
EgIDA6_S	CAAGCGGAGAACTTCTTTGG
EgIDA6AS	GTGCTGTATAAATTGCCACCTTC
EgIDA7_S	AGCAAGCAGATGGCAAACTC
EgIDA7_AS	ATTTGCTTGCCTCATTCTCG
EgIDA10_S	TATCCCGAAGATGGCAGTTC
EgIDA10_AS	GAAAGAAAAGAAAGCGTTCCAA
PtIDA_S	GACCGGGCGCTACTCTAATGG
PtIDA_AS	TGGATGGACCAGAAGGTGGAA
PtIDL1_S	TGTTCCACCTTCGGCTCCAT
PtIDL1_AS	CCACTTGGTGGCCATGTCTG
Pt019G078400_S	GGGAAGCTGCCTGAGGGAAT
Pt019G078400_AS	GTGAGAACCGGCCGAAATTG
Pt007G135400_S	CTTTGCCGCCTGACCAATCT
Pt007G135400_AS	CGGACAGTGACTCGGGAAGG
PtACTIN1_S	CGATGCCGAGGATATTCAAC
PtACTIN1_AS	ACCAGTGTGTCTTGGTCTACCC
