# Supplementary figures and images for: Conservation of the abscission signaling peptide IDA during Angiosperm evolution: withstanding genome duplications and gain and loss of the receptors HAE/HSL2
Source: Front Plant Sci. 2015 Oct 30;6:931. doi: 10.3389/fpls.2015.00931 (PMC4627355; doi:10.3389/fpls.2015.00931)

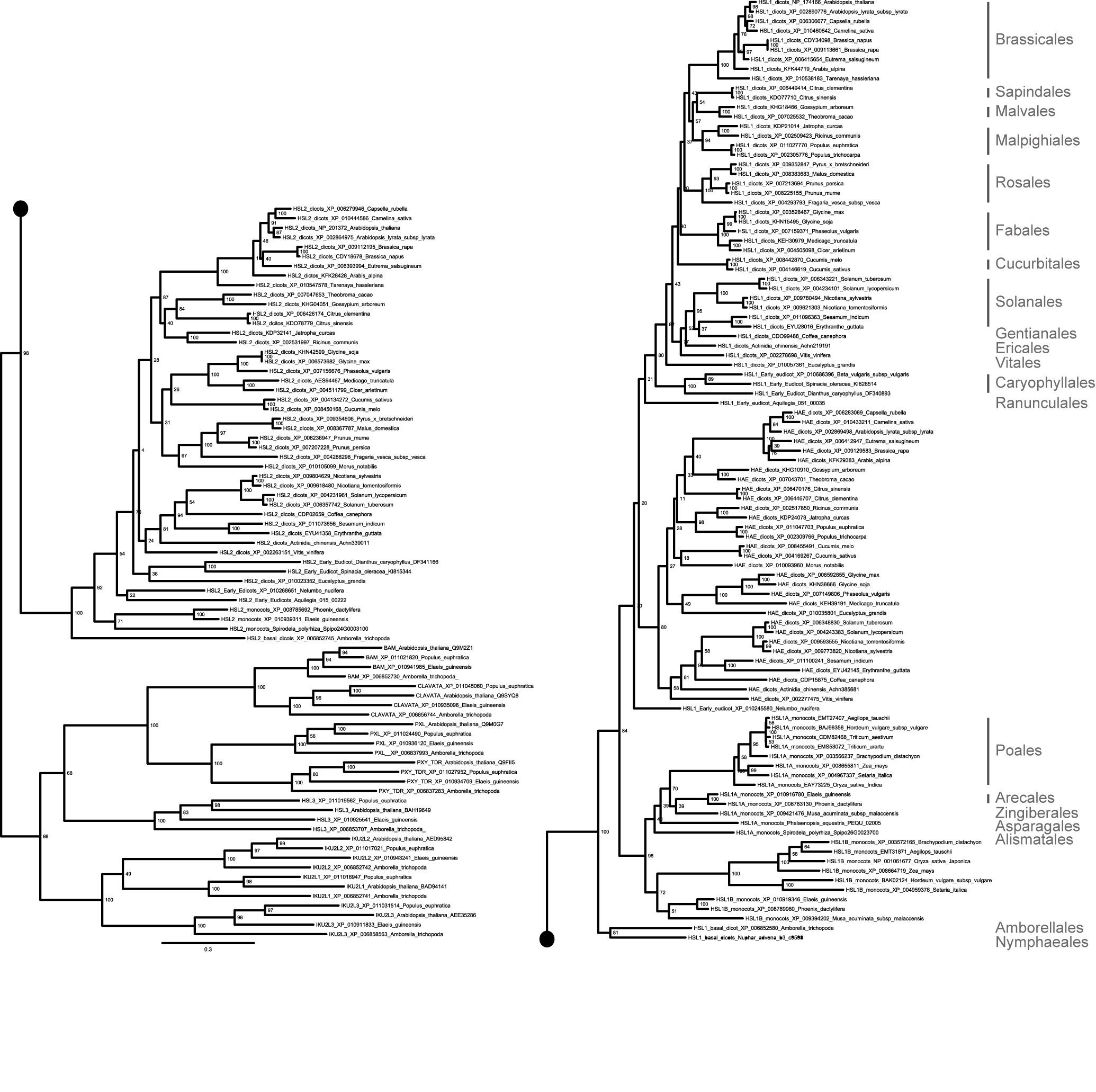

Supplement: Supplementary file 5 [file Image1.TIF]

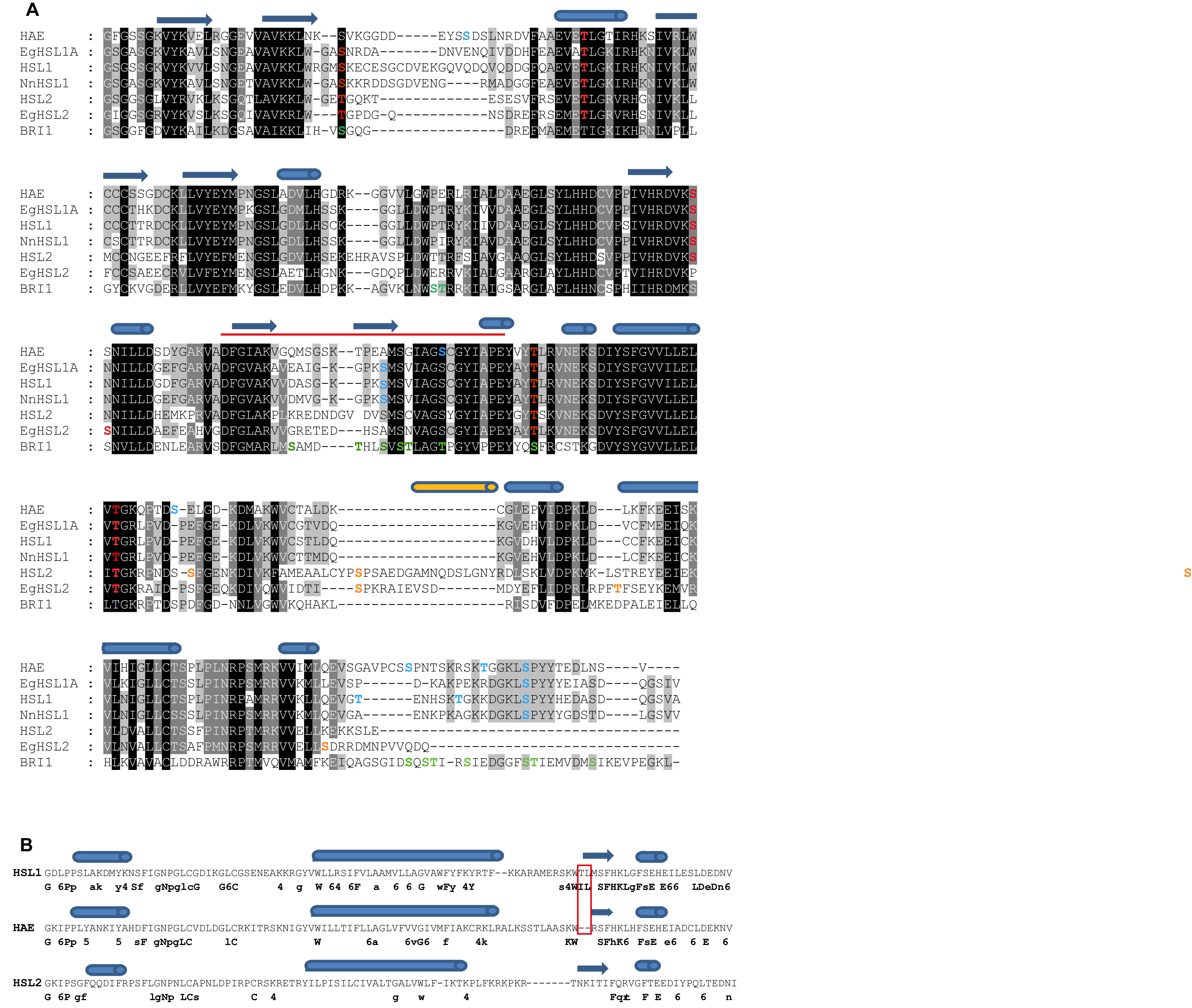

Supplement: Supplementary file 6 [file Image2.TIF]

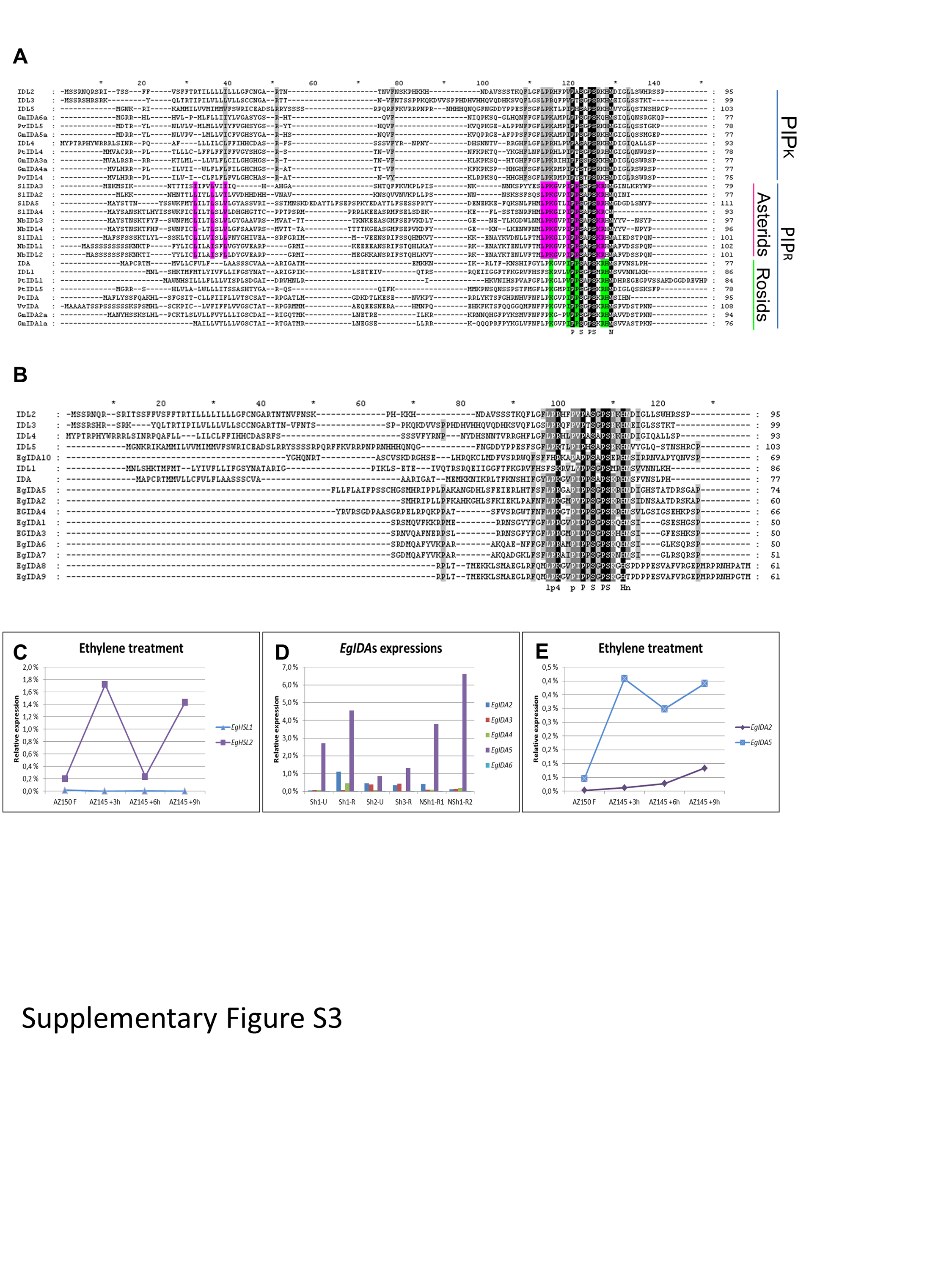

Supplement: Supplementary file 7 [file Image3.TIF]
